# Evidence of extraganglionic vagal mechanoreceptors in the mouse vagus nerve

**DOI:** 10.1111/joa.13925

**Published:** 2023-07-05

**Authors:** Luis Leon‐Mercado, Arely Tinajero, Laurent Gautron

**Affiliations:** ^1^ Department of Internal Medicine Center for Hypothalamic Research, UT Southwestern Medical Center Dallas Texas USA

**Keywords:** anatomical variation, autonomic nervous system, interoception, neuroanatomy, rodent

## Abstract

Vagal afferent neuronal somas are in the nodose and jugular ganglia. In this study, we identified extraganglionic neurons in whole‐mount preparations of the vagus nerves from Phox2b‐Cre‐ZsGreen transgenic mice. These neurons are typically arranged in small clusters and monolayers along the cervical vagus nerve. Although infrequent, these neurons were sometimes observed along the thoracic and esophageal vagus. We performed RNAscope in situ hybridization and confirmed that the extraganglionic neurons detected in this transgenic mouse strain expressed vagal afferent markers (i.e., *Phox2b* and *Slc17a6*) as well as markers that identify them as potential gastrointestinal mechanoreceptors (i.e., *Tmc3* and *Glp1r*). We also identified extraganglionic neurons in the vagus nerves of wild‐type mice that were injected intraperitoneally with Fluoro‐Gold, thereby ruling out possible anatomical discrepancies specific for transgenic mice. In wild‐type mice, extraganglionic cells were positive for peripherin, confirming their neuronal nature. Taken together, our findings revealed a previously undiscovered population of extraganglionic neurons associated with the vagus nerve. Going forward, it is important to consider the possible existence of extraganglionic mechanoreceptors that transmit signals from the abdominal viscera in future studies related to vagal structure and function.

## Graphical abstract

We identified extraganglionic vagal afferent neurons in whole‐mount preparations of the vagus nerves from Phox2b‐Cre‐ZsGreen transgenic mice. These neurons are typically arranged in small clusters and monolayers along the cervical vagus nerve (X), far from the nodose ganglion (NG).

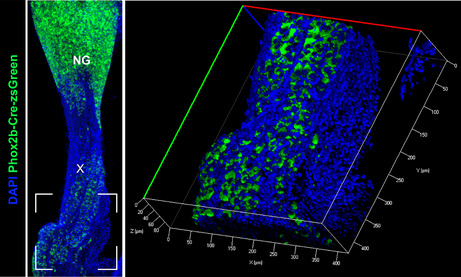

## 1 INTRODUCTION

The vagus nerve contains nerve fibers emanating from vagal sensory and motor neurons (Fox et al., 2001; Havton et al., 2021; Mei et al., 1980; Stakenborg et al., 2020; Zhao et al., 2022). Anatomical sites innervated by the vagus nerve include, but are not limited to, the heart, lung, diaphragm, and most organs of the abdominal cavity (Wang et al., 2017). Previous findings reported in most textbooks and review articles state that afferent neuronal somas of the mammalian vagus nerve are located in the nodose and jugular ganglia (NG and JG, respectively) (Berthoud & Neuhuber, 2000; Li & Schild, 2002; Mazzone & Undem, 2016; Niu et al., 2020; Prescott & Liberles, 2022; Zhao et al., 2022). However, early anatomical studies described a population of cells that resemble neurons in the histological preparations of vagus nerve tissue from cats, dogs, humans, and newly hatched chicks (Plenat et al., 1988). In their 1988 study, Plenat and colleagues (Plenat et al., 1988) confirmed the existence of human cells that were “…indistinguishable in shape and histochemical properties from those constituting the inferior vagal ganglion and which were scattered along the trunk or formed microscopic ganglia”. They also reported that the neurons in the vagus nerve represented between 1%–2% of all vagal sensory neurons. More recent histological studies identified a population of sparse pseudo‐unipolar cells that resembled vagal afferent neurons in cross‐sections of the porcine vagus nerve (Pelot et al., 2020; Settell et al., 2020) and rat whole vagal trunk (Wang et al., 2019). Whereas Plenat and colleagues (Plenat et al., 1988) described these neurons as “displaced sensory neurons*”*, in this study, we have identified vagal afferent neurons with soma outside the NG and JG as *extraganglionic neurons*. While the aforementioned observations suggest that the vagus nerve may contains extraganglionic vagal afferent neurons, there are very few published studies that address this point in rodents. Thus, and to the best of our knowledge, the existence of extraganglionic neurons in rodent species remains unclear. In this study, we explored this point using a whole‐mount approach and fluorescent reporter mice and identified previously unreported population of extraganglionic vagal afferent neurons with anatomical features consistent with abdominal mechanoreceptors.

## 2 METHODS

### 2.1 Mice and sample preparation

Paired‐like homeobox 2b (Phox2b)‐Cre mice (stock# 016223; RRID:IMSR_JAX:016223) and ZsGreen reporter mice (stock# 007906; RRID:IMSR_JAX:007906) were obtained from the Jackson Laboratory. Mice were cross‐bred to generate strain that carried one Phox2b‐Cre allele and one floxed‐STOP‐ZsGreen allele (Phox2b‐Cre‐ZsGreen mice). Vagal neurons associated with the NG were fluorescently labeled (i.e., ZsGreen‐positive) in these mice. Fifteen male Phox2b‐Cre‐ZsGreen and seven male wildtype (WT) mice (Jackson Laboratory, stock: 000664 | B6, C57BL/6J) were used in this study. All procedures were approved by the University of Texas Southwestern Medical Center at Dallas Institutional Animal Care and Use Committee. Mice were housed in a light‐controlled (12 h on/12 h off; lights on at 07:00 h) and temperature‐controlled (21.5–22.5°C) barrier facility. On the day of sacrifice, each mouse was anesthetized by intraperitoneal chloral hydrate (500 mg/kg) followed by transcardial perfusion with saline and 10% formalin (Sigma). Ganglionic masses attached to the vagus nerve were carefully removed using a dissecting scope and fine forceps.

### 2.2 Whole‐mount imaging

Samples from Phox2b‐Cre‐ZsGreen mice were prepared for whole‐mount imaging following a protocol adapted from Dodt and colleagues (Dodt et al., 2007). Briefly, the dissected vagal ganglionic mass from Phox2b‐Cre‐ZsGreen mice was placed in 10% formalin for 1 h. at room temperature, rinsed in phosphate‐buffered saline (PBS), and then incubated for 10 min with 4′,6‐diamidino‐2‐phenylindole (DAPI) (ACD, RNAscope multiplex kit cat. 323,110). Samples were then dehydrated in a graded ethanol series (50%, 70%, and 100%) for 1 h. each. Samples were then stored in 100% ethanol overnight at room temperature. Dehydrated samples were transferred into a clearing solution that consisted of one part benzylalcohol (Sigma) and two parts benzyl benzoate (Sigma) and incubated for at least 2 days at room temperature before imaging. While clearing by this method resulted in a significant shrinkage of our preparations and some loss of fluorescence (data not shown), the ZsGreen signal remained bright enough to facilitate our evaluation of vagal neuron topography by confocal microscopy. Cleared samples used for whole‐mount preparations were imaged using a Zeiss microscope LSM880 confocal laser scanning microscope. Images were collected using the following parameters: 2048 × 2048; average of 8; zoom of 1; 10× objective with 0.3 numerical aperture; step of 1.5 μm; power laser adjusted to 0.61 and 1 (optional correction in z); gain of 600; and 1.56 as the refractive index correction. Up to 200 optical sections were acquired per channel (i.e., ZsGreen and DAPI).

### 2.3 Multiplex fluorescence in situ hybridization (ISH)

We performed RNAscope ISH to visualize select markers of different subsets of vagal afferents on thin tissue sections of the NG and vagus nerve as described in our earlier publication (Bob‐Manuel & Gautron, 2021). Among selected markers were *Phox2b* (all NG neurons), *Slc17a6* (all NG and JG neurons), *Tmc3* (subsets of mechanoreceptors), *Glp1r* (gastric mechanoreceptors), *Agtr1*(subsets of mechanoreceptors), TH (subsets of mechanoreceptors), and *Scn10a* (all nociceptive neurons) (for details see results and [Bai et al., 2019; Kupari et al., 2019; Zhao et al., 2022]). After perfusion and dissection, samples were transferred to 30% sucrose solution and incubated for 24 h, before freezing on a bed of dry ice without additional clearing or processing. Samples from Phox2b‐Cre‐ZsGreen mice were cut to 14 μm thickness using a cryostat. Because there are comparatively few extraganglionic neurons in these preparations, we generated sections from the entire cervical vagus nerve from each mouse. Reagents and probes used for RNAscope ISH are listed in Table 1. Details of the procedure were reported in our previous publication (Bob‐Manuel & Gautron, 2021). Following RNAscope, we performed immunostaining for tyrosine hydroxylase (TH). Briefly, slides were incubated overnight with a 1:2000 dilution of a primary antibody directed against TH ([Bookout & Gautron, 2021]; Table 1; [Aves Labs Cat# TYH, RRID:AB_10013440]). Slides were washed and incubated with secondary antibodies and fluorophore (1:1000 dilution) for 1 h. All preparations were counterstained with DAPI and mounted with ProLong Gold Antifade mounting medium (Invitrogen, cat#P36934). ZsGreen fluorescence could be detected clearly after RNAscope ISH without additional immunostaining. Tissues sections subjected to RNAscope were imaged by confocal microscopy (Zeiss LSM880) using the following parameters: 1024 × 1024; average of 8; zoom of 1; 20× or 63× objectives; step of 1.5 μm; power laser adjusted to 1 and 2; and a gain ranging from 700 to 850. False colors were applied to each channel to optimize visual contrast. For cell counting, digital images of the NG and vagus nerve were collected using a Leica DM6 B microscope with a DFC900GT camera. Neurons that were positive for both ZsGreen and selected markers were manually counted on the digital images. Fluorescence was confirmed by eye rather than by threshold intensity considering that RNAscope generated virtually no background staining. Data are presented as the percent of ZsGreen neurons counted in the NG and vagus nerve preparations.

**TABLE 1:** List of antibodies and probes used for immunohistochemistry and multiplex in situ hybridization, respectively.

Antibody (manufacturer)	Cat# (lot#)	Concentration
TH antibody (Aves lab)	TYH (TYH6917982)	1:2000
Peripherin antibody (Abcam)	Ab1530	1:500
Biotin‐SP AffiniPure Donkey Anti‐Chicken (Jackson Immunoresearch)	703,065,155 (161856)	1:1000
Streptavidin, Alexa Fluor™ 488 Conjugate (Invitrogen)	S32354 (2387462)	1:1000
Streptavidin Alexa Fluor™ 594 (Invitrogen).	S32356	1:1000

RNAscope probe from ACD	Cat#	
*Scl17a6*	319,171‐C2	1:50
*Tmc3*	426,301	1:1
*Agtar1*	481,161‐C2	1:50
*Scn10a*	426,011‐C2	1:50
*Glp1r*	418,851	1:1
*Phox2b*	407,861‐C2	1:50

### 2.4 Peripherin immunofluorescence

Samples from two male wild‐type (WT) mice were prepared as described earlier. Tissue sections were incubated overnight with rabbit anti‐peripherin primary antibody (see Table 1 for details), washed three times with PBS, followed by 1 h incubation with a Biotin SP conjugated donkey anti‐rabbit antibody (Jackson Immunoresearch cat# 711‐065‐152; 1/1000). Then, slides were washed three times with PBS and incubated in Streptavidin Alexa Fluor 594 (Invitrogen cat# S32356; 1/1000). Slides were washed three times with PBS and mounted in Vectashield antifade mounting media with DAPI (Vector laboratories cat# H1200) with a coverslip. Images were acquired with confocal microscopy.

#### 2.4.1 Fluoro‐Gold tracing

To verify if the extraganglionic neurons observed in Phox2b‐Cre‐ZsGeen mice are also present in wild type mice and rule out anatomical changes associated with genetically modified animals, we used retrograde tracing. Fluoro‐Gold (FG; Fluorochrome) was dissolved in sterile saline. In one group (*n* = 4), <200 μL of a 1% FG solution was administered to each mouse by intraperitoneal route (i.p.). Using this method, FG ultimately reaches the bloodstream and will label most (potentially all) neurons with projections outside the blood–brain barrier (Berthoud & Powley, 1996). Mice in a second group (*n* = 2) were anesthetized with isoflurane and prepared aseptically for surgery. The abdominal organs were exposed via a vertical incision; 30 μL of a 0.025% FG solution was applied directly to the surface of the stomach. Using this method, FG will label subsets of neurons that are preferentially connected to the abdominal viscera (Leon Mercado et al., 2019; Sterner et al., 1985). Upon completion of the FG application, the peritoneum and skin were closed with sutures. 3–4 days later, all mice that received FG were perfused as described before. The NG and vagus nerve were extracted, submerged in PBS, and imaged as a whole‐mount sample. Imaging was performed with a Leica DM6 B microscope equipped with an FG filter immediately after sample collection.

### 2.5 Acquisition, processing, and presentation of digital images

ImageJ Fiji (U.S. National institutes of Health; RRID:SCR_003070) was employed to merge z‐stacks and convert digital images to a TIFF format. Contrast, brightness, and resolution (300 dpi) of all digital images were adjusted uniformly in Adobe Photoshop 2021; this software was also used to add scale bars and arrange our images into annotated plates. LASX was used to capture digital images and count RNAscope‐ and FG‐labeled cells. ZEN was used to collect high‐resolution images of cleared preparations and tissues evaluated by RNAscope for illustration purposes.

Lastly, subgroups of four Phox2b‐Cre and four FG‐treated mice were used to estimate the number of extraganglionic cells in the vagus nerve and their position relative to the NG. Briefly, the right and left vagi were collected after perfusion, post‐fixed 4 h and washed with PBS. Nerves were then transferred to PBS/glycerol and mounted on slides. Whole‐mount imaging was done using a DM6B‐Z microscope (Leica) 20X and photomicrographs were merged using Leica application suite X. Images were analyzed using ImageJ version 2.9.0/1.53 t. Counted the number of individual positive cells visible along each nerve and their distance relative to the peripheral edge of the NG.

## 3 RESULTS

### 3.1 The mouse vagus nerve contains extraganglionic neurons

**FIGURE 1:**
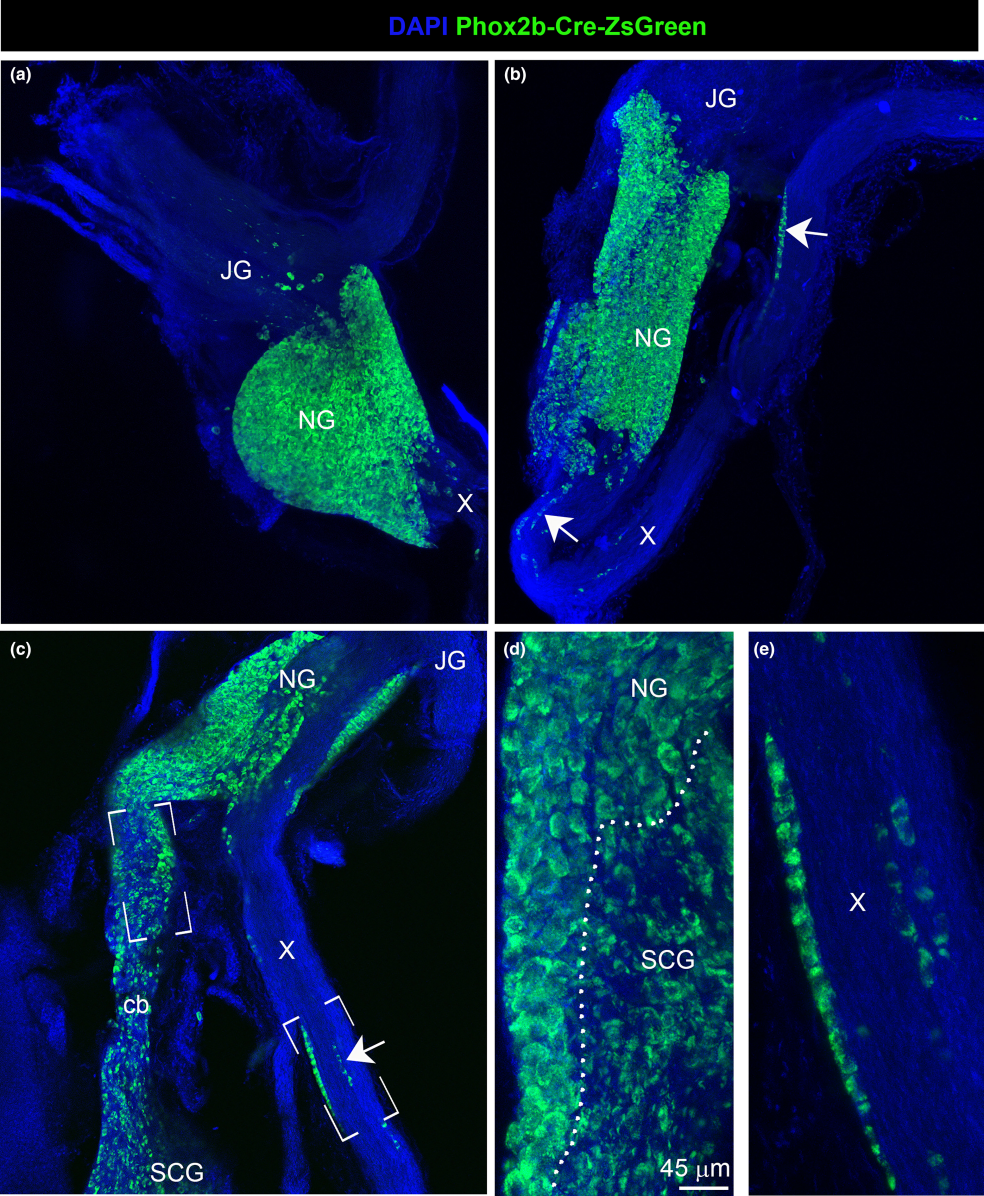
Whole‐mount preparations of the vagal ganglionic mass reveals the existence of extraganglionic neurons in Phox2b‐Cre‐ZsGreen mice. All samples from Phox2b‐Cre‐ZsGreen mice were counterstained with DAPI, cleared, and imaged with confocal microscopy. (a) Bright fluorescence was detected in Phox2b‐positive cell bodies of vagal afferent neurons in the bulb‐like shape NG, but not in the JG. (b) In this sample, Phox2b‐positive cell bodies are distributed in an elongated shape. Cells in the vagus nerve are identified with white arrows. (c) an illustration of a cell bridge connecting the NG to the SCG; neurons in the vagus nerve and SCG are ZsGreen‐positive. The insets show the locations of the higher magnification views displayed in D and E. (d) A ZsGreen‐positive cell bridge connecting NG and SCG with both vagal afferent neurons and sympathetic neurons. The fluorescence signal is somewhat less intense in the SCG compared to NG (transition demarcated by dotted line). (e) Scattered ZsGreen‐positive cells in the vagal trunk and at the peripheral edge of the vagus nerve. Extraganglionic cells in the vagus nerve are often found in monolayers. cb, cell bridge; JG, jugular ganglion; NG, nodose ganglion; SCG, superior cervical ganglion; X, vagus nerve. Scale bar in (a) applies to (b) and (c). Scale bar in (d) applies to (e).

**FIGURE 2:**
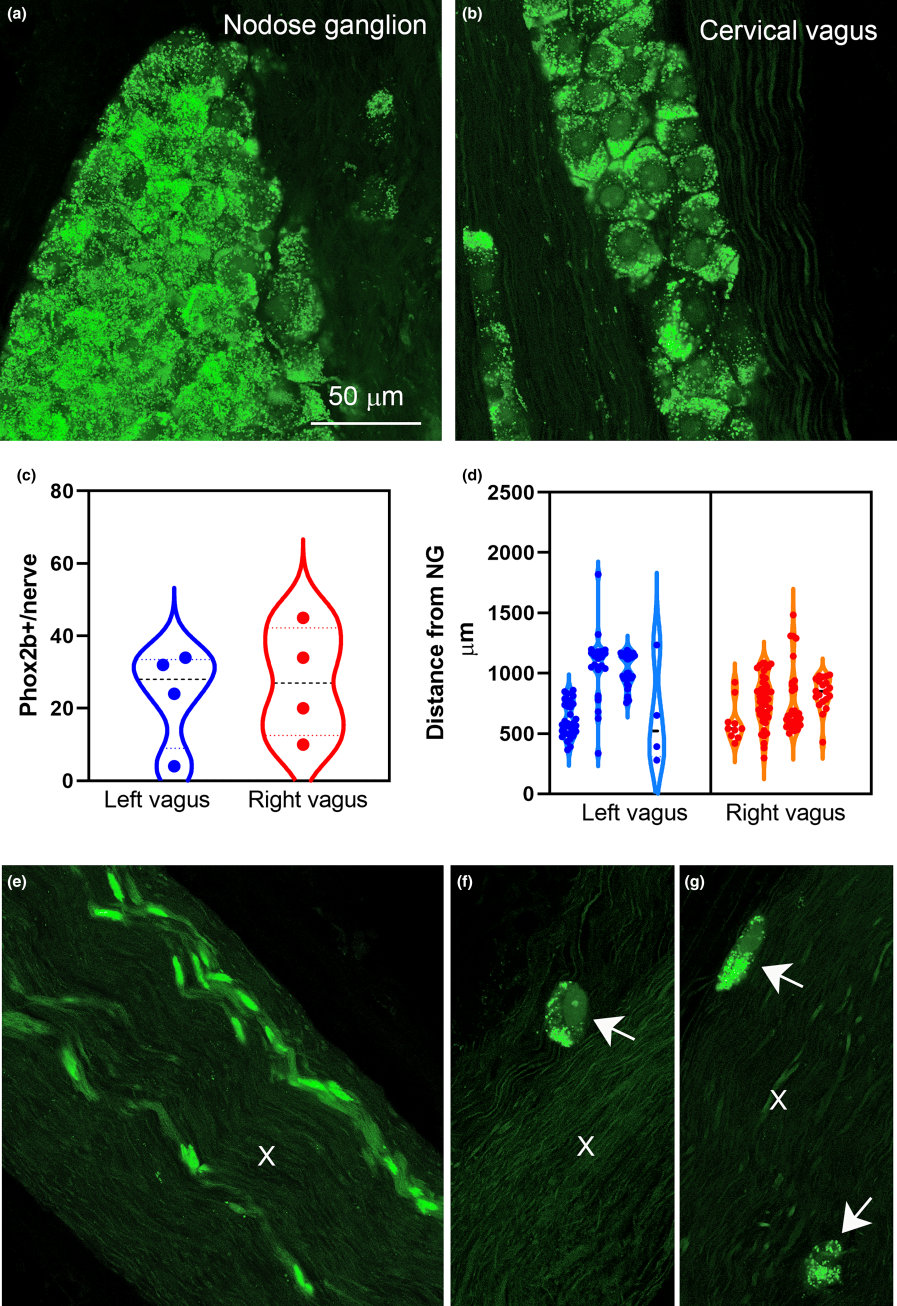
Distribution of extraganglionic neurons along the vagus nerve of Phox2b‐Cre‐ZsGreen mice in whole‐mount preparations. (a) High magnification view of ZsGreen‐positive cells in the NG and (b) along the cervical vagus nerve. The extraganglionic neurons arranged in cell monolayers resemble vagal afferent neurons. (c) Estimates of the number of ZsGreen‐positive neurons visible in the left and right vagus nerves of four mice. (d) Estimates of the distance (μm) separating individual extraganglionic ZsGreen neurons and the peripheral edge of the NG. (e) Examples of scattered ZsGreen‐positive cells resembling glial cells. Isolated ZsGreen‐positive neurons observed in the thoracic (f) and esophageal (g) vagus nerve (white arrows). NG, nodose ganglion. Scale bar in (a) applies throughout.

In all resected samples, vagal neurons in the NG emitted intense green fluorescence in whole‐mount preparations of the vagus nerve from Phox2b‐Cre‐ZsGreen mice. Phox2b is a transcription factor important in the development of the autonomic nervous including sensory neurons of the nodose ganglion (Pattyn et al., 1999). Phox2b‐positive vagal neurons were detected in 19 samples (11 left and 8 right side) dissected from 14 different male transgenic mice; no signals were detected in the JG (Figure 1a). The NG display a typical bulb‐like shape in 79% of the samples examined (Figure 1a) and was more elongated forming like a sleeve around the vagus nerve in another 21% (Figure 1b). Interestingly, brightly‐labeled cells were detected in extraganglionic sites within the vagus nerve itself (Figure 1b). As described in our previous publication (Bookout & Gautron, 2021), the NG is connected by a cell bridge to the superior cervical ganglia (SCG) in 21% of these samples (Figure 1c,d). SCG neurons were also ZsGreen‐positive (Figure 1d); this observation is consistent with previous reports that documented the expression of Phox2b mRNA in post‐ganglionic sympathetic neurons (Bookout & Gautron, 2021; Grillet et al., 2003). All samples examined contained extraganglionic cells in varying numbers (Figure 1e). Typically, these extraganglionic cells formed small clusters within the nerve itself or, more often, generated monolayer that were detected at the periphery of the vagus nerve (Figures 1b,e and 2a,b). At higher magnification, ZsGreen‐positive cells located in the vagal trunk resembled pseudo‐unipolar neurons in terms of shape and size (Figure 2a,b). In a subset of animals (*n* = 4), we estimated the number of extraganglionic neurons to be approximately 30 per nerve, with a range of 4–45 (Figure 2c,d). There was no difference between the left and right sides. In a subset of animals (*n* = 4), the average distance of extraganglionic neurons from the NG was approximately 862 μm, with a range of 280–1818 μm, with no difference between the left and right sides (Figure 2c,d). Occasionally, patches of ZsGreen‐positive resembling glial cells (small and elongated) were also observed along the vagus nerve (Figure 2e). The identity of these cells is uncertain, but PHOX2B signaling is known to occur in glial cells (Espinosa‐Medina et al., 2014). In addition, while extraganglionic neurons were detected primarily within the cervical vagus nerve in a region below the NG, isolated zsGreen neurons could occasionally be seen at distal sites within the thoracic and esophageal vagus nerve (Figure 2f,g); however, these cells were rare and not consistently observed.

### 3.2 Vagal extraganglionic neurons are enriched in mechanoreceptors markers

**FIGURE 3:**
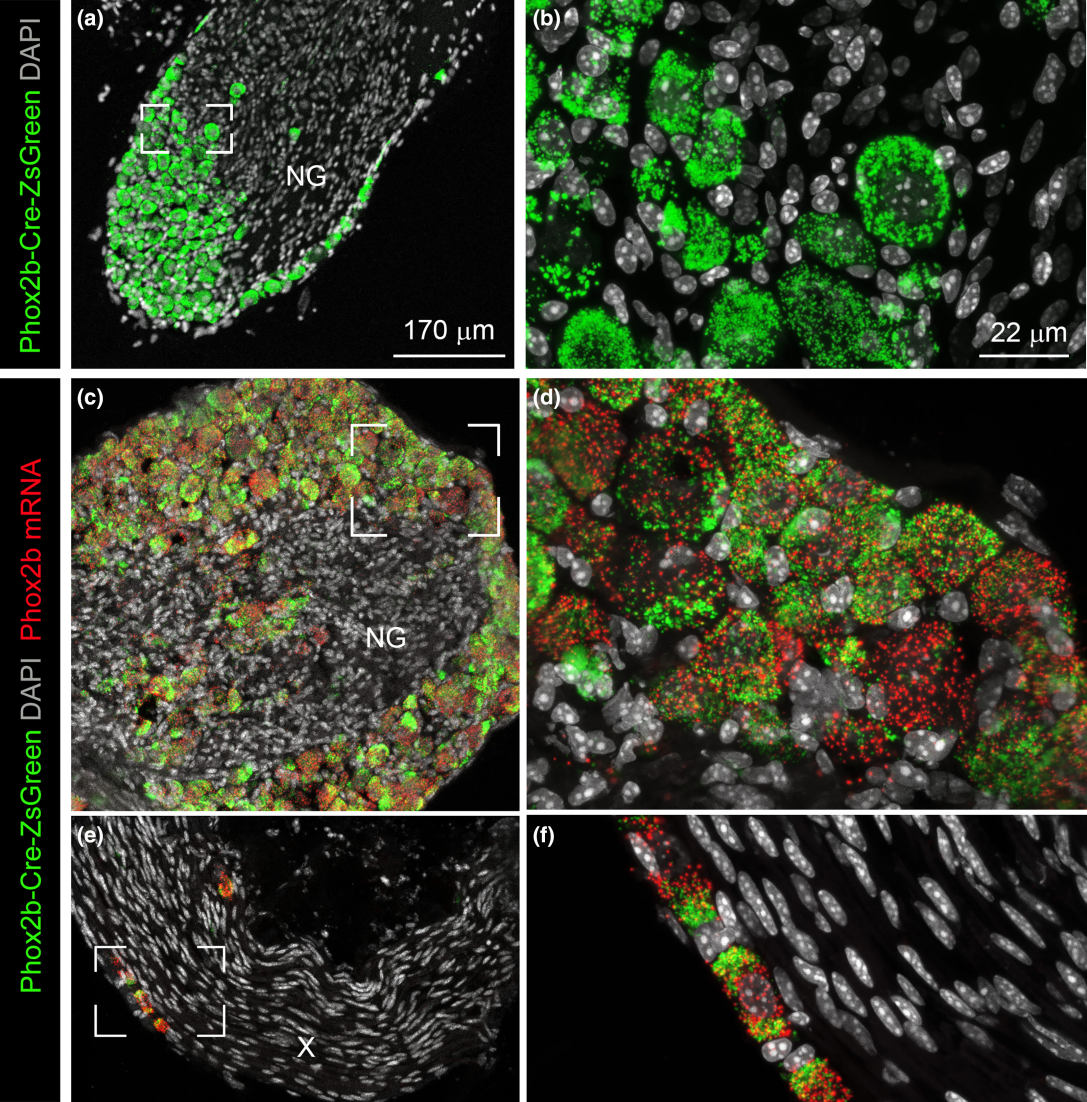
Validation of *Phox2b* mRNA expression of by vagal neurons of Phox2b‐Cre‐ZsGreen mice. Fluorescent RNAscope in situ hybridization was performed on samples from Phox2b‐Cre‐ZsGreen mice to confirm Phox2b mRNA expression in extraganglionic cells. Images were obtained by confocal microscopy of thin sections of the NG or vagus nerve following RNAscope in situ hybridization. Tissues were counterstained with DAPI (shown in grey for better contrast). (a, b) All the neurons in the NG showed bright ZsGreen fluorescence. (c, d) RNAscope in situ hybridization validated extensive expression of *Phox2b* mRNA (red dots) by ZsGreen cells. (e, f) RNAscope in situ hybridization to detect *Phox2b* mRNA (red dots) in the vagus nerve. All ZsGreen‐positive extraganglionic cells expressed Phox2b mRNA. Scale bar in (a) applies to (c) and (e). Scale bar in (b) applies to (d) and (f).

**FIGURE 4:**
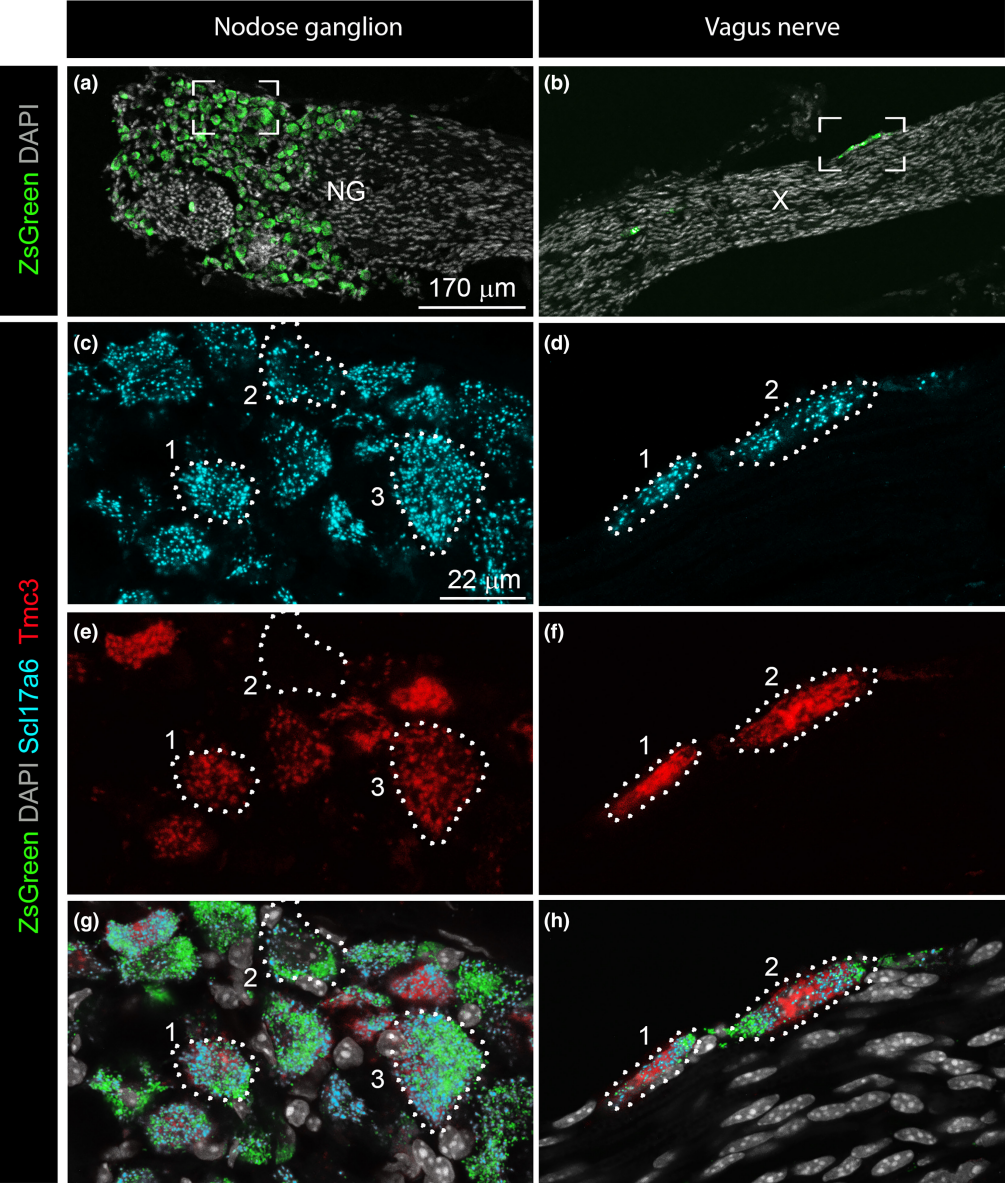
Extraganglionic vagal cells express *Tmc3* and *Slc17a6*. Fluorescent RNAscope in situ hybridization was performed on samples from Phox2b‐Cre‐ZsGreen mice to visualize the expression of Tmc3 and Slc17a6 mRNA. Images were obtained by confocal microscopy of thin sections of the NG (left) or vagus nerve (right) following RNAscope in situ hybridization. Tissues were counterstained with DAPI (shown in grey for better contrast). (a, c, e, g) All ZsGreen‐positive neurons in the NG expressed *Slc17a6* (dotted outlines 1, 2, and 3). A large subset of ZsGreen‐positive neurons in the NG expressed *Tmc3*; neurons shown in profiles 1 and 3, but not profile 2, expressed *Tmc3*. (b, d, f, h) Extraganglionic neurons identified in the vagus nerve expressed both *Slc17a6* and *Tmc3*, (e.g., dotted outlines in profiles 1 and 2). Scale bar in (a) applies to (b). Scale bar in (c) applies to (d–h).

**FIGURE 5:**
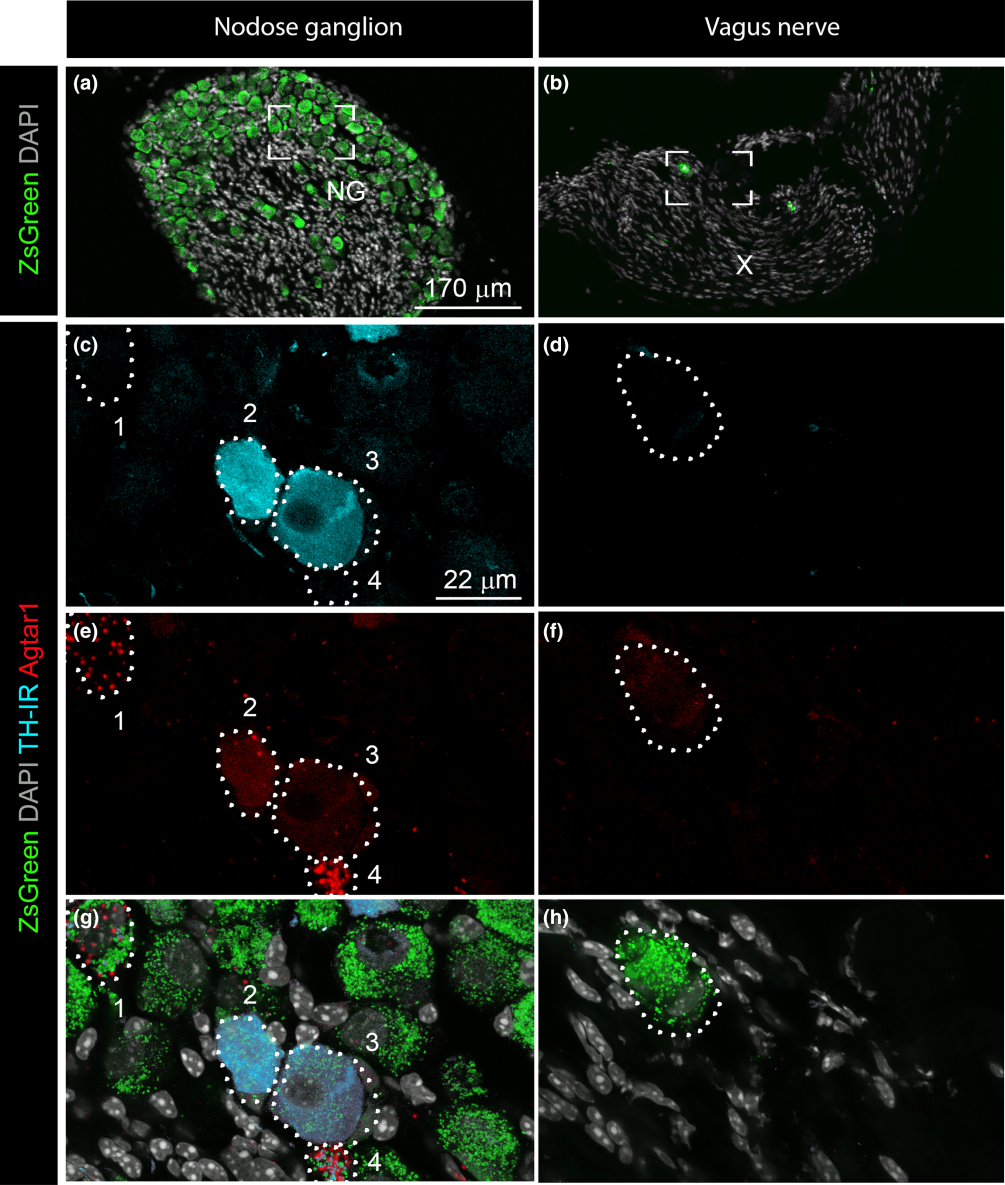
Extraganglionic neurons in the vagus nerve do not express tyrosine hydroxylase or *Agtar1*. Fluorescent Immunohistochemistry and RNAscope in situ hybridization was performed on samples from Phox2b‐Cre‐ZsGreen mice to visualize the presence if TH and *Agtar1* mRNA respectively. Confocal microscopy images of thin sections of the NG (left) or vagus nerve (right) were obtained following RNAscope in situ hybridization. Tissues were counterstained with DAPI (in grey for better contrast). (a, c, e, g) Immunoreactivity for TH and *Agtar1* mRNA expression varied between neurons in the NG. For example, we detected *Agtar1* but not TH in profiles 1 and 4 (dotted outlines 1 and 4). By contrast, both TH and *Agtar1* were detected in profile 2. Profile 4 only produced TH. (b, d, f, h) Neither marker was detected in extraganglionic neurons in the vagus nerve (dotted outline). Scale bar in (a) applies to (b). Scale bar in (c) applies to (d–h).

**FIGURE 6:**
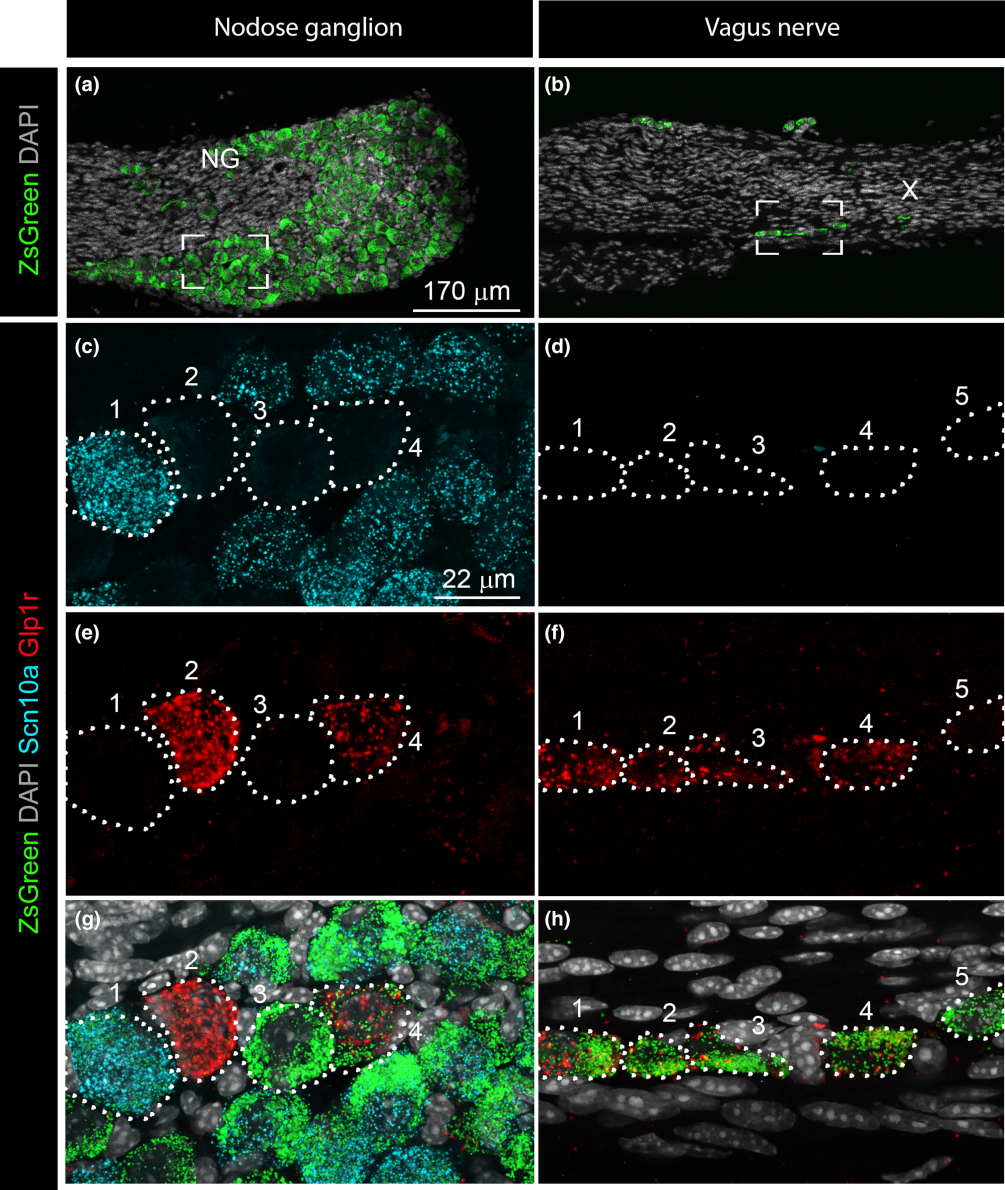
Extraganglionic neurons frequently express *Glp1r*. Fluorescent RNAscope in situ hybridization was performed on samples from Phox2b‐Cre‐ZsGreen mice to visualize *Glp1r* and *Scn10a* mRNA expression in extraganglionic cells. Confocal microscopy images were obtained from thin sections of the NG (left) or vagus nerve (right) following RNAscope in situ hybridization. Tissues were counterstained with DAPI (shown in grey for better contrast). (a, c, e, g) While a large subset of neurons in the NG expressed *Scn10a*, a smaller, distinct subset expressed *Glp1r*. For example, profiles 2 and 4 expressed high levels of *Glp1r* but not *Scn10a* (dotted outlines). (b, d, f, h) While most extraganglionic neurons express *Glp1r* (profiles 1, 2, 3, and 4), none express *Scn10a*. Scale bar in (a) applies to (b). Scale bar in (c) applies to (d–h).

Tissue sections from the Phox2b‐Cre‐ZsGreen mice were used to label vagal neurons in combination with RNAscope ISH. ZsGreen fluorescence was preserved after ISH without the need of immunostaining; and most NG neurons appeared with bright green dots (Figure 3a,b). As anticipated, ISH signals for *Phox2* mRNA overlapped with those of ZsGreen within the NG (Figure 3c,d; Table 2) and the vagus nerve itself (Figure 3e,f; Table 2). ZsGreen‐positive neurons in the NG extensively expressed high levels of *Slc17a6* (Figure 4a,c,e,g; Table 2), which is a marker of vagal sensory neurons (Bookout & Gautron, 2021; Kupari et al., 2019; Zhao et al., 2022). Importantly, extraganglionic Phox2b‐expressing neurons were also positive for *Slc17a6*, thereby confirming their identity as vagal afferent neurons (Figure 4b,d,f,d,h; Table 2). *Tmc3* expression was assessed as a marker of mechanoreceptors (Zhao et al., 2022). In all samples, *Tmc3* was detected in a large subset of NG neurons (Figure 4a,c,e,g; Table 2) and extraganglionic neurons (Figure 4a,c,e,g). We also assessed the expression of mechanoreceptors markers tyrosine hydrolase (TH), angiotensin II receptor type 1 (*Agtr1*), and glucagon‐like peptide1 receptor (*Glp1r*) (Bai et al., 2019; Kupari et al., 2019; Williams et al., 2016; Zhao et al., 2022). Interestingly, TH and *Agtr1* were detected in a subset of NG neurons (Figure 5a,c,e,g; Table 2), but not extraganglionic neurons (Figure 5a,c,e,g; Table 2). By contrast, *Glp1r* expression was detected in neurons in the NG and the vagus nerve (Figure 6a–h; Table 2). *Scn10a* encodes for a sodium channel, voltage‐gated, type X alpha subunit (Na_v_1.8) which is enriched in wide range of unmyelinated or lightly myelinated vagal chemoreceptors (Kupari et al., 2019). Whereas most NG neurons were *Scn10a*‐positive, we identified no *Scn10a*‐positive extraganglionic neurons (Figure 6a,c,e,g). Taken together, these expression patterns suggest that extraganglionic vagal neurons are afferent in nature and represent mechanoreceptors (Bai et al., 2019; Kupari et al., 2019; Williams et al., 2016; Zhao et al., 2022).

**TABLE 2:** Markers co‐expressed by ZsGreen‐positive neurons in the nodose ganglion (NG) versus vagus nerve.

Markers combination	ZsGreen + Phox2b
NG (100 profiles)	99.5 ± 0.6%
Vagus nerve (20 profiles)	100.0 ± 0.0%

Markers combination	ZsGreen + Slc17a6	ZsGreen + Tmc3
NG (383 profiles)	98.5 ± 0.5%	70.5 ± 4.4%
Vagus nerve (83 profiles)	98.5 ± 0.5%	100.0 ± 0.0%

Markers combination	ZsGreen + TH‐IR	ZsGreen + Agtar1
NG (524 profiles)	7.5 ± 2.4%	17.0 ± 3.6%
Vagus nerve (55 profiles)	0.0 ± 0.0%	0.0 ± 0.0%

Markers combination	ZsGreen + Scn10a	ZsGreen + Glp1r
NG (578 profiles)	73.8 ± 6.2%	18.0 ± 5.6%
Vagus nerve (23 profiles)	3.3 ± 2.2%	73.8 ± 6.8%

*Note*: Mean percentages ± Standard deviation; (*n* = 4 ganglia per combination) of ZsGreen‐positive profiles co‐expressing select markers of vagal neurons within the NG relative to the vagus nerve itself (extraganglionic neurons). The total number of sampled profiles for each combination is indicated in parentheses. Due to the scarcity of extraganglionic neurons, the number of counted profiles in the vagus nerve was always smaller than in the NG. Coexpression patterns were obtained based in multiplex RNAscope and, in the case of TH, RNAscope combined with immunostaining.

### 3.3 Vagal extraganglionic neurons are connected to abdominal viscera

**FIGURE 7:**
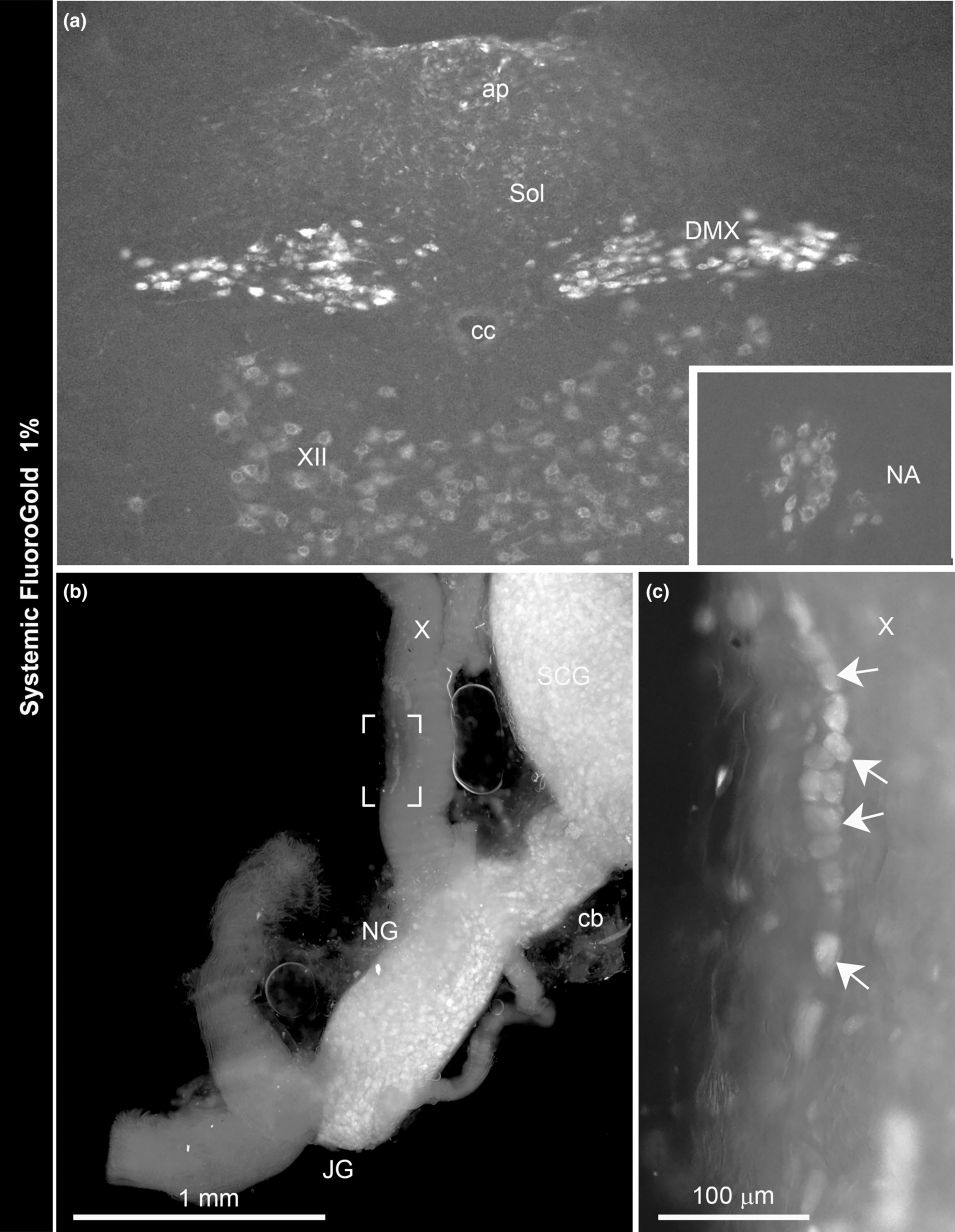
Intraperitoneal administration of FG labels extraganglionic neurons in Wild‐type mice. Retrograde tracing was used to rule out possible anatomical discrepancies between transgenic Phox2b‐Cre and wild‐type mice. (a) Brainstem neurons, including those contributing to the motor component of the vagus nerve, were labeled in mice that underwent an intraperitoneal injection of 1% FG. The inset in the bottom right corner represents FG‐labeled cells in the nucleus ambiguus. Images were obtained from a thin section of the brainstem of a WT C57Bl6/J mouse. (b) A whole‐mount preparation from the same mouse revealed that nearly all sensory neurons in the vagal ganglionic complex were also FG‐labeled. (c) Clusters of FG‐labeled cells were also detected within the vagus nerve (white arrows). The extraganglionic cells resembled previously‐described ZsGreen positive afferent neurons. In this sample, neurons located in the SCG and cell bridge connecting it to the NG were also labeled with FG. FG labeling is shown in black and white for better contrast. ap, area postrema; cb, cell bridge; cc, central canal; DMX, dorsal motor nucleus of the vagus; JG, jugular ganglion; NA, nucleus ambiguus; NG, nodose ganglion; Sol, solitary nucleus; XII, hypoglossal nucleus; X, vagus nerve.

**FIGURE 8:**
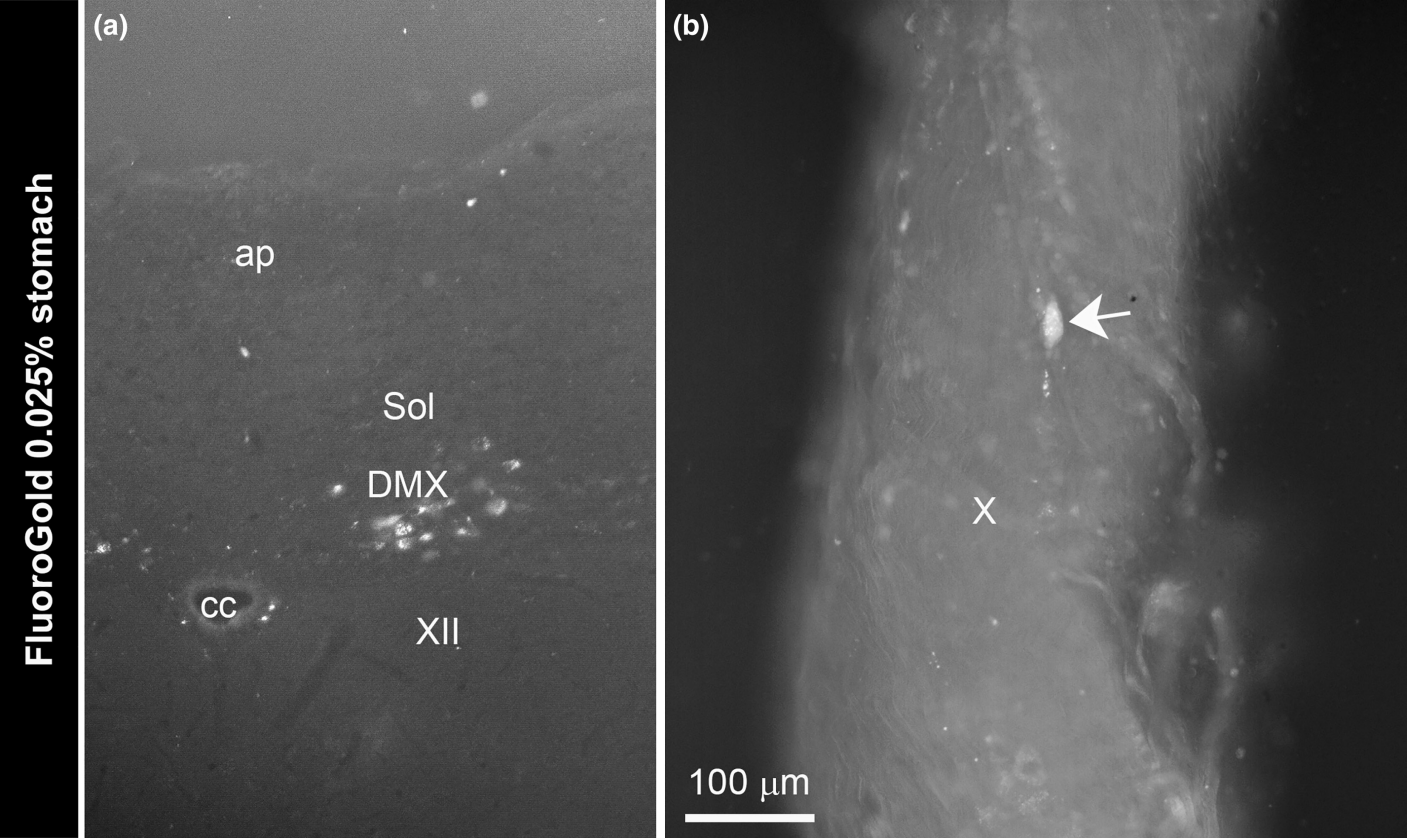
Direct administration of FG to the stomach wall labels extraganglionic neurons in Wild‐type mice. (a) A few brainstem neurons were labeled in response to the direct application of diluted FG to the surfaces of the stomach. Among these neurons were those that preferentially innervated abdominal organs. We detected FG‐labeling of a subset of neurons in the DMX, but none in the XII. This image was obtained from a thin section of the brainstem of a wild‐type C57BL/6J mouse. (b) While very few FG‐labeled cells were detected in the vagus nerve in a whole‐mount preparation from the same mouse (white arrow), their topography and shape were similar to previous descriptions of extraganglionic neurons. For abbreviations see legend to Figure 7.

**FIGURE 9:**
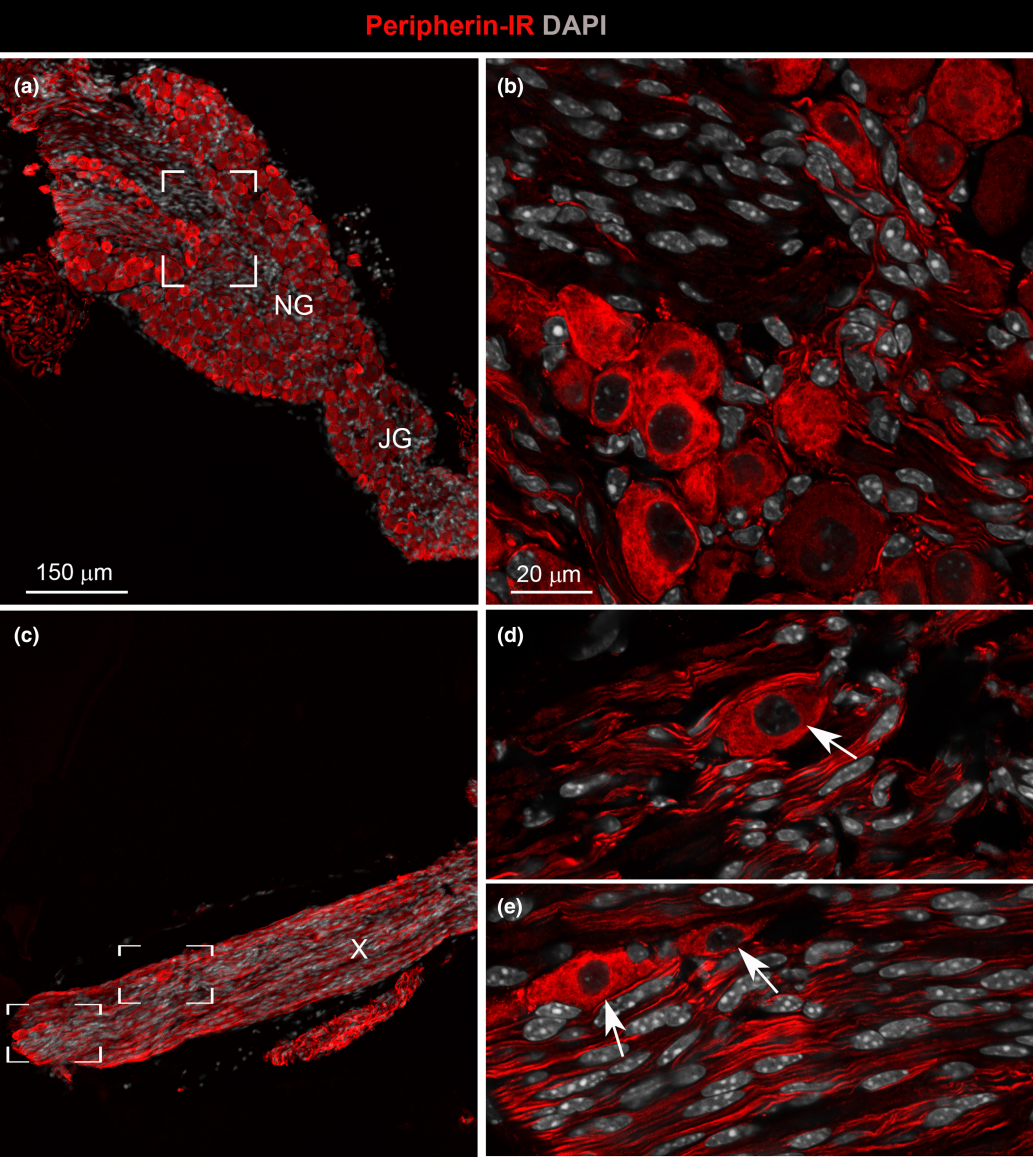
Peripherin immunoreactivity in tissue sections of the vagus nerve of wild‐type mice. (a, b) In the NG, all neurons and their axons were labeled with peripherin immunoreactivity. (c–e) On tissue sections of the cervical vagus nerve, isolated peripherin‐positive neurons were observed (white arrows). The distribution and shape of the latter neurons is highly consistent with that of Phox2b‐positive extraganglionic neurons described before. NG, nodose ganglion; JG, jugular ganglion; X, vagus nerve.

Our next experiments were designed to verify that extraganglionic neurons were not the result of abnormal vagus nerve development in mice carrying the Phox2b‐Cre and/or ZsGreen transgenes. First, we investigated the vagus nerves of wild‐type male C57Bl6/J mice treated with FG (Berthoud & Powley, 1996). When FG is injected in the periphery, it is taken up by neurons and travels retrogradely along their axons to label the cell bodies of neurons located outside the blood–brain barrier. In the first experiment, in which mice underwent intraperitoneal administration of FG, fluorescence was detected in all brainstem neurons with projections outside the blood–brain barrier including the dorsal motor nucleus of the vagus, the hypoglossal and nucleus ambiguus neurons (Figure 7a). Their vagus nerves were also examined in whole‐mount preparations. While the level of fluorescence was not nearly as bright as ZsGreen, FG administration resulted in fluorescence from nearly all neurons in the NG and SCG (Figure 7b). Of eight examined nerves, two showed no FG‐labeled extraganglionic staining. However, we observed FG‐positive extraganglionic neurons in the vagus nerves of the six remaining nerves (Figure 7c). We estimated the number of FG‐extraganglionic neurons to be approximately seven per nerve, with a range of 1–22. The average distance of FG‐extraganglionic neurons from the NG was approximately 695 μm, with a range of 20–2940 μm. Although fewer cells were observed using FG compared to Phox2b, the distribution and shape of FG‐labeled extraganglionic neurons were consistent with the properties described previously for ZsGreen‐tagged neurons. In other experiments, we administered a small amount of FG directly onto the stomach wall via a method that was used previously by us and others (Leon Mercado et al., 2019; Sterner et al., 1985). FG was detected in a small subset of neurons in the dorsal motor nucleus of the vagus but was not detected in hypoglossal neurons (Figure 8a). FG‐labeled neurons were detected in the NG and, more importantly, in the cervical vagus nerve (Figure 8b). The presence of FG‐labeled cells was verified only qualitatively. Thus, the tracing data confirm the existence of extraganglionic neurons exist in WT mice and suggest that they are likely to be connected to abdominal viscera. Lastly, we used peripherin immunostaining to confirm the neuronal identity of the cells located in the vagus nerve of wild‐type male C57Bl6/J mice. Intense peripherin immunoreactivity was observed in the somas and axons of vagal afferents in the NG (Figure 9a,b). On tissue sections of the vagus nerve, occasionally, isolated peripherin‐positive neurons were observed along the cervical vagus nerve itself (Figure 9c–e). The distribution, shape, and size of peripherin‐stained neurons were entirely consistent with that of the extraganglionic cells previously described.

## 4 DISCUSSION

This study identified and described extraganglionic vagal afferent neurons in the mouse vagus nerve. Small clusters of specialized sensory cells known as vagal paraganglia are embedded in the vagus nerve of many animal species including the mouse (Goormaghtigh, 1936; Plenat et al., 1988; Prechtl & Powley, 1985). Vagal paraganglia generally form ellipsoidal structures embedded in the nerve trunk. In addition, vagal paraganglia cells closely resemble glomus cells of the carotid body (small prismatic shape) as opposed to neurons; thus, the extraganglionic neurons observed in our study were unlikely to be components of vagal paraganglia. In addition, paraganglionic cells are TH‐positive (Goehler et al., 1997). Our results revealed that extraganglionic neurons are sparsely distributed along the vagus nerve which may explain why there is so little literature on this subject. Currently, we are unaware of any descriptions of vagal extraganglionic neurons in the mouse. Here, we identified and localized these neurons in whole‐mount samples and studied their distribution along the vagus nerve by means of thin sections. Extraganglionic neurons can also be seen on thin tissue sections of the vagus nerve if it has been carefully dissected and entire nerve sections are produced. Our anatomical and molecular studies revealed that vagal extraganglionic neurons were most likely a type of gastrointestinal mechanoreceptors. We found that extraganglionic neurons expressed *Tmc3* and *Glp1r*, which are genes enriched in neurons projecting to the gastrointestinal tract (Williams et al., 2016; Zhao et al., 2022). Our experiments using FG as a retrograde tracer also indicate that these extraganglionic neurons potentially innervate the abdominal cavity. We sought to label neurons connected to peritoneal organs rather than specifically targeting the stomach (or any other organs) following the Sterner et al. (1985) protocol. Of note, whereas the intraperitoneal (i.p.) approach resulted in labeling of the XII and nucleus ambiguus, the peritoneal application did not. This indicates the specificity of the intra‐abdominal route and suggests that the tracer did not reach thoracic organs in significant amounts. Moreover, early anatomical and electrophysiological studies revealed that vagal afferent neurons are arranged in the NG in “a crude topographical representation of the alimentary tract” (Altschuler et al., 1989; Mei, 1970). Vagal afferents to the gastrointestinal tract tend to be concentrated in the lower half of the NG. Thus, it is logical to assume that vagal afferents below the NG might be connected to abdominal organs rather than those in the thorax.

Based on the limited number of markers assessed in our study, it is also difficult to predict precisely which type of endings correspond to extraganglionic neurons. However, previous studies have reported Glp1r to be particularly abundant in gastric intraganglionic endings (Williams et al., 2016, Zhao et al., 2022). Nonetheless, we do not know whether *Tmc3* and *Glp1r* are completely absent from mechanoreceptors innervating tissues in the thoracic cavity. Thus, it cannot be rule out that subsets of extraganglionic neurons may also be connected the carotid and lungs which are anatomical sites highly enriched in mechanoreceptors of vagal origin (Wang et al., 2017). At the central level, considering that Glp1r‐positive vagal afferent neurons are known to project into the NTS (Zhao et al., 2022), it is very likely that extraganglionic neurons may be connected to the NTS. Alternatively, these neurons may connect the spinal trigeminal and paratrigeminal nuclei as well (Neuhuber & Berthoud, 2022). Further research aiming to visualize neuronal circuits in whole animals must be done in the future to trace selectively the projections of these neurons from peripheral organs to the brainstem.

If a similar distribution applies to humans as well, this could carry implications and their presence should be investigated. The neurons identified by Plenat and colleagues described as “displaced sensory neurons*”* (Plenat et al., 1988) are most likely the same structures as the vagal extraganglionic neurons described in our current study. There is currently strong interest in the use of vagus nerve stimulation (VNS) as a method to treat depression, epilepsy, and inflammatory diseases (Bonaz et al., 2019; Chavan et al., 2017). In therapeutic VNS procedures, electrodes are typically attached to the cervical vagus nerve. If extraganglionic neurons exist in humans, as implied by early anatomical studies (Plenat et al., 1988), electrode leads placed nearby might compromise their integrity. For instance, one previous study performed in rats revealed inflammation around the vagus nerve near the site of cuffing (Somann et al., 2018). The complexity of the structure of the human vagus nerve surpasses that of rodents. Prior anatomical and modeling investigations have indicated that the placement of electrodes in various locations surrounding the human vagus nerve may engage distinct groups of fascicles, resulting in diverse physiological effects. In addition, determining the position of extraganglionic neurons relative to the electrode placement may prove valuable in anticipating the side effects and outcomes of vagus nerve stimulation. It is also possible that certain stimulatory parameters might activate these neurons more easily.

Previous studies, including our own, have overlooked the presence of extraganglionic neurons. These neurons may represent a previously unknown population of vagal neurons with unique connectivity, biochemical make‐up, and functions. Going forward, it is important to consider the existence of these neurons in studies related to the vagus nerve. Finally, it is also important to note that Wetmore and Elde have previously described small sensory ganglia in rats, composed of neurons with nociceptive morphological features, that are associated with the spinal accessory nerve rather than the dorsal root ganglion (Wetmore & Elde, 1991). Notably, these neurons formed scattered ganglia that were adherent to the spinal accessory nerves, which closely resemble the description of vagal extraganglionic neurons. These observations suggest that sensory neurons with an extraganglionic position may be more common than previously known and may be associated with nerves other than the vagus nerve.

## AUTHOR CONTRIBUTIONS

Luis Leon‐Mercado contributed to the design, data interpretation, and approval of the article. Arely Tinajero specifically contributed to Fluoro‐Gold studies. Laurent Gautron contributed to all other aspects including the acquisition of histological data and drafting of the manuscript.

## CONFLICT OF INTEREST STATEMENT

The authors have no conflicts of interest to disclose.

## Data Availability

The data that support the findings of this study including cell counts and original digital images are available from the corresponding author upon reasonable request.
